# Traditional fermented foods and beverages in Iraq and their potential for large-scale commercialization

**DOI:** 10.1186/s42779-022-00133-8

**Published:** 2022-05-18

**Authors:** Belal J. Muhialdin, Viachaslau Filimonau, Jamal M. Qasem, Salam A. Ibrahim, Hussein L. Algboory

**Affiliations:** 1 Department of Food Science and Nutrition, University of Minnesota, 1334 Eckles Ave, Saint Paul, MN 55108 USA; 2 University of Surrey, Stag Hill, Guildford, GU2 7XH UK; 3 Hotelschool The Hague, Brusselselaan 2, 2587 AH Den Haag, The Hague, The Netherlands; 4 Agriculture Directorate of Naynawa, Ministry of Agriculture, 41001 Mosul, Nainawa Iraq; 5 Food and Nutritional Sciences Program, North Carolina A&T State University, Greensboro, NC 27411 USA; 6 Faculty of Food Science, Al-Qasim Green University, Babylon, Iraq

**Keywords:** Mesopotamia, Traditional food, Heritage cooking, Fermentation, Health benefits

## Abstract

The tradition of making fermented foods and beverages in Iraq dates back to 7500 BC. These fermented foods and beverages are represented by meat-, milk-, vegetable-, and fruit-based products reflecting diversity of agricultural production in ancient Iraq (Mesopotamia). Although the recipes for some fermented foods and beverages were lost throughout history, those remaining foods and beverages occupy a noticeable position in modern Iraqi cuisine. In this review, knowledge and techniques for preparation of 5 traditional fermented foods, i.e. *Basturma*, Smoked *Liban,* *Aushari* cheese, *Turshi*, and Sour *Khobz,* and 3 fermented beverages, i.e. *Shanina*, *Sharbet Zbeeb*, and *Erk Sous* in Iraq, are documented. Traditional fermented foods and beverages have multiple health benefits because of high content of probiotics and bioactive compounds. Traditional fermented foods and beverages are made using the back-slopping technique which ensures safety of production and maintains organoleptic properties. The review highlights the potential of fermented foods and beverages for their large-scale commercialization.

## Introduction

Iraq is historically known as the Mesopotamia, the region formed between the rivers of Tigris and Euphrates, often referred to as a cradle of ancient civilizations [1]. Due to its long history, Iraq has rich cultural heritage reflected in diversity of its national cuisine [2]. The Old Testament highlights cuisines of the Babylonian and Assyrian Empires represented by hundreds of types of bread, porridge, and cheese [2]. The Sumerian Empire established in (what is now) southern Iraq was one of the first civilizations to make cheese known as the “*Ga-har*” [3]. The Sumerians prepared a unique beverage called the “beer bread” from germinated barley seeds which was purified to obtain a liquid known as the “*sikura*” [4]. The Sumerians documented bread fermentation and baking processes. Hundreds of the discovered clay tablets demonstrated the knowledge that was established by the Sumerian Empire on fermented foods as a source of daily diets among its residents [5].

Fermented foods and beverages were integral to many ancient diets due to pleasant taste compared to the raw materials of which these fermented foods and beverages were made. Fermented foods represented an important source of nutrients in a time of crisis due to their extended shelf life. For example, the study of the most recent crisis in Iraq confirmed the importance of traditional fermented foods in sustaining nutrition of Iraqi households in critical times [6]. The preparation techniques of fermented foods and beverages were transferred from generation to generation over the thousands of years, thus making these foods and beverages traditional [7].

Traditional fermented foods and beverages are part of multiple cultures, and some of these foods and beverages have been produced on a large scale. For example, *Turshi*, fermented mixed vegetables, including cucumbers, bell pepper, cabbage, and garlic, is an important element of modern Iraqi cuisine [8]. *Basturma,* seasonal fermented lamb minced meat, is a popular winter foodstuff in Iraq that is consumed because of high protein and energy content [9]. Similarly, a fermented beverage, such as *Shanina* (yogurt beverage), is consumed in Iraq daily and *Sherbet Zabeeb* (fermented raisin) is consumed during the Ramadan.

Although fermented foods and beverages have traditionally been consumed because of their pleasant taste, the biotechnology analysis demonstrates significant health benefits associated with consumption of traditional fermented foods and beverages. These benefits are associated with the presence of microorganisms known as probiotics, including bacteria (*Lactobacillus, Bifidobacterium, Streptococcus, Leuconostoc, Pediococcus, Propionibacterium, Bacillus, and Enterococcus*) and fungi (*Saccharomyces, Aspergillus, and Candida*) [10]. In addition, some bioactive compounds released during the fermentation process make fermented foods and beverages an important source of peptides, *γ*-aminobutyric acid (GABA), fatty acids, amino acids, and vitamins [11].

Fermentation involves two techniques, i.e. spontaneous fermentation and back-slopping fermentation. Spontaneous fermentation is performed by mixing the raw material with brine, and fermentation is done by several microorganisms associated with this raw material. Back-slopping fermentation is carried out by mixing the ingredients with subsequent sterilization required to deactivate the microorganisms associated with the raw material. Following this, a small amount of previously fermented ingredients is added to the sterilized ingredients and further fermented at room temperature. According to Li and Gänzle [12], back-slopping fermentation ensures safety of final product and can improve food quality due to the presence of stable microflora.

Fermented foods and beverages have been popular with consumers who consider these foods and beverages important for the immune system, especially during COVID-19 [13]. Traditional fermented foods and beverages of Iraq have not been commercialized because of low public awareness of their existence alongside health benefits. This review aims to document traditional fermented foods and beverages in Iraq and explain their preparation techniques to preserve these foods and beverages for future generations. The commercialization potential of traditional fermented foods and beverages in Iraq will also be briefly discussed.

## The region of Mesopotamia (Iraq)

**Fig. 1:**
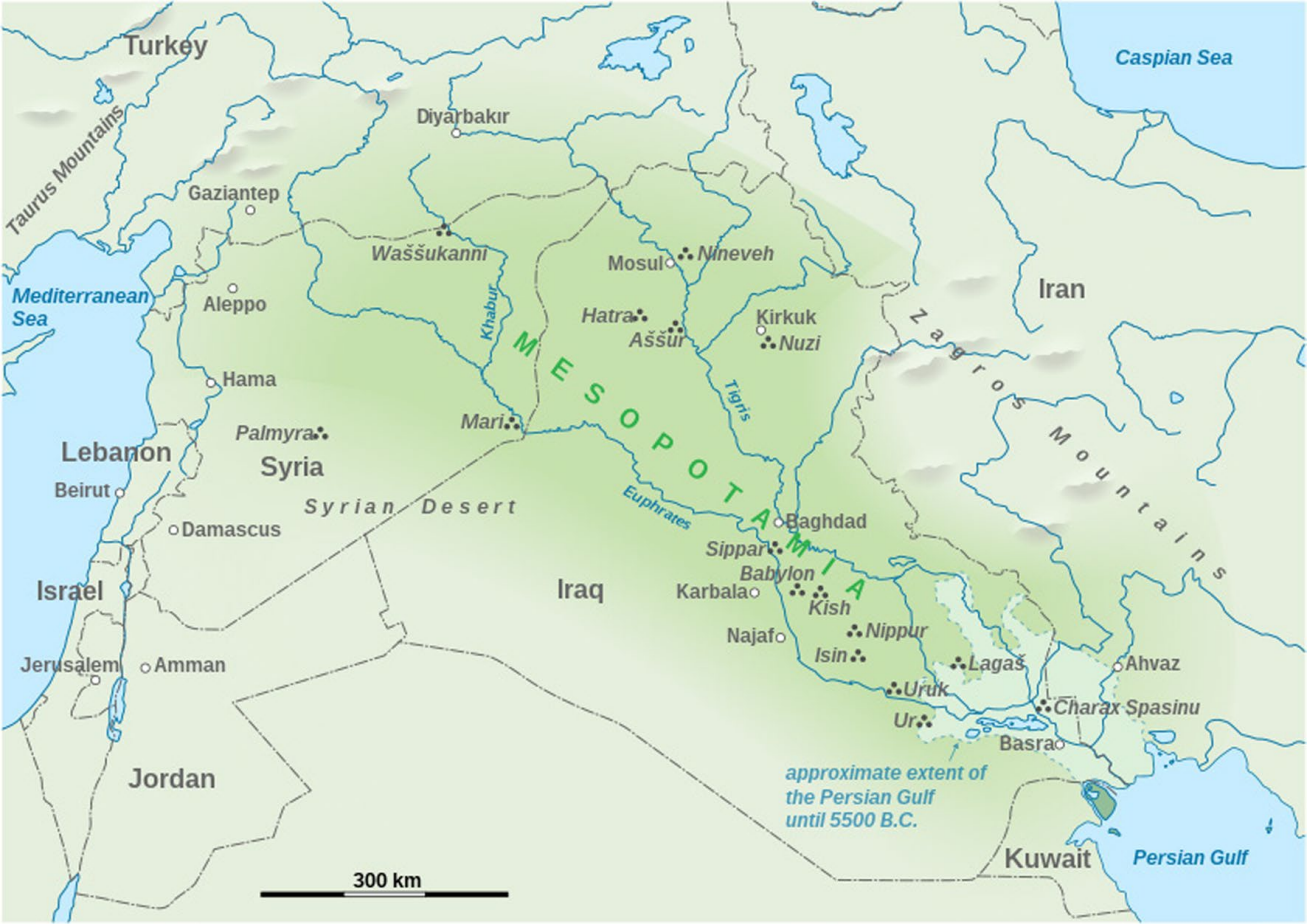
Mesopotamia map know as modern Iraq ( Source: Wikimedia)

Iraq (Mesopotamia) is a region in the Middle East with hot and dry summers (43 °C at day time, 26 °C at night time) and moderate winters (16 °C at day time, 2 °C at night time) which is due to its climate ranging from the Mediterranean to hot semi-arid continental [14]. Iraq is positioned between 33.2232° N latitude and 43.6793° E longitude (Fig. 1). The range of temperatures has determined diversity of cultivated vegetables and fruits in Iraq. Wheat cultivation dominates in the North of Iraq given the origin of wheat from the *Fertile Crescent* region in northern Iraq [15]. In the South of Iraq, date fruits are mainly cultivated and believed to have originated from this region [16]. Iraq is home to several ethnic races with the majority belonging to the Arabs, and several minorities including the Kurds, Turkmen, Assyrians, Yazidis, and Shabak [17]. The rich ethnic mixture influenced the variety and affected diversity of traditional fermented foods and beverages in Iraq.

## Methods

This ethnographic research is based on traditional knowledge of fermented foods and beverages that was transferred in Iraq from generation to generation. Primary data were collected following the method described by Fontefrancesco [18], with some modifications applied. The data were collected from May 2020 to December 2020 in cities of Mosul (*Basturma*, *Turshi, Sharbet Zbeeb, and Erk Sous*), Erbil (Smoked *Liban*), Sulaymaniyah (*Aushari* cheese), Baghdad (Sour *Khobz, Shanina (Shineena)*), Babil (*Turshi Medabis*), and Al Muthana (*Turshi Medabis*). Traditional fermented foods and beverages were first studied through the literature review; this desk-based research was then supplemented with field observations and interviews administered with local small-scale producers and households. A total of 11 small-scale producers (third to fifth generation) and 8 households (women aged 58–64) were interviewed in this study. The interviews documented traditional preparation methods, mode, and time of consumption and examined the sociocultural significance of traditional foods and beverages in Iraq. Information about the current opportunities and challenges of making and distributing traditional fermented foods and beverages was collected from small-scale producers.

## Traditional fermented foods

Traditional fermented foods in Iraq are regionally diverse due to the rich history of the country. For example, in the ancient city of Babylon, fermented foods are derived from date (*Phoenix dactylifera* L) fruits. The ancient city of Nineveh, a former capital of the Assyrian Empire, is known for fermented foods prepared from vegetables, wheat, and barley. In the southern city of Basrah located in the vicinity of the Arabian Gulf, traditional fermented foods are prepared from fish, such as mackerel (*Scomber scombrus*) and cobia (*Rachycentron canadum*). The preparation techniques are another source of diversity of Iraqi fermented foods as they show significant cross-regional differences. This section will highlight the preparation methods and the raw materials applied to the reviewed traditional fermented foods in Iraq.

### Basturma

**Fig. 2:**
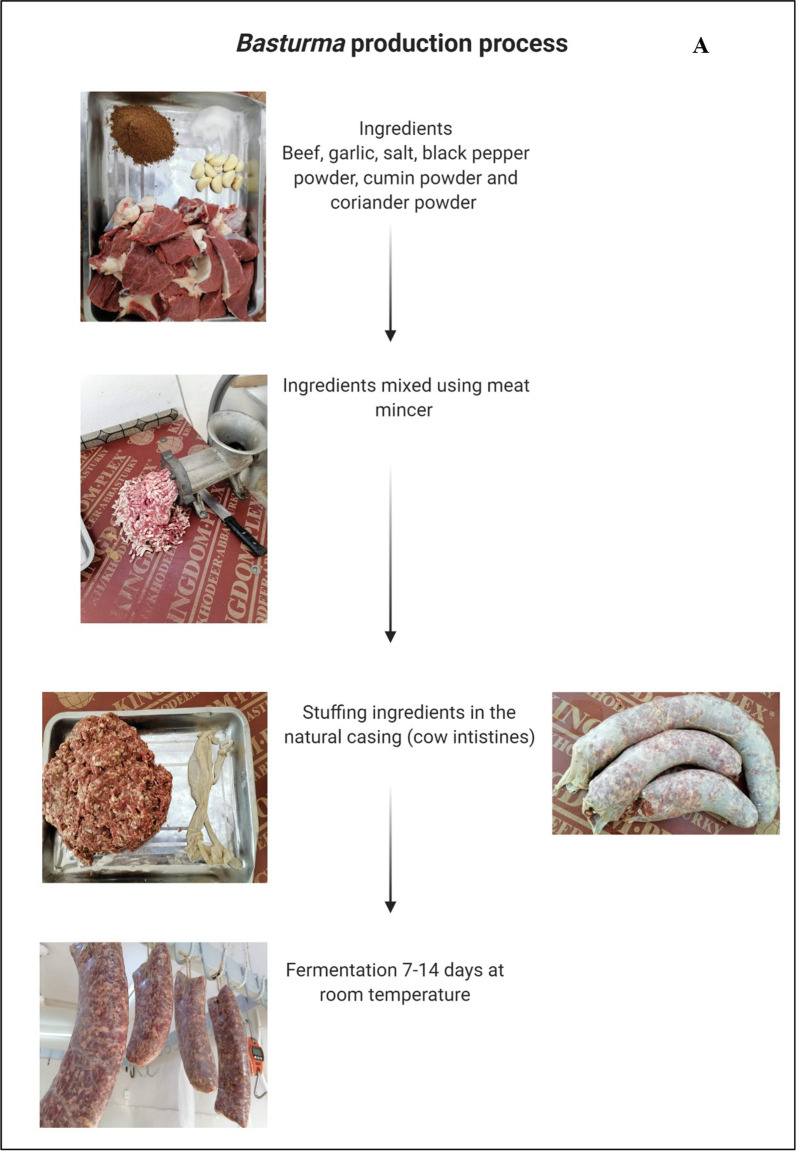

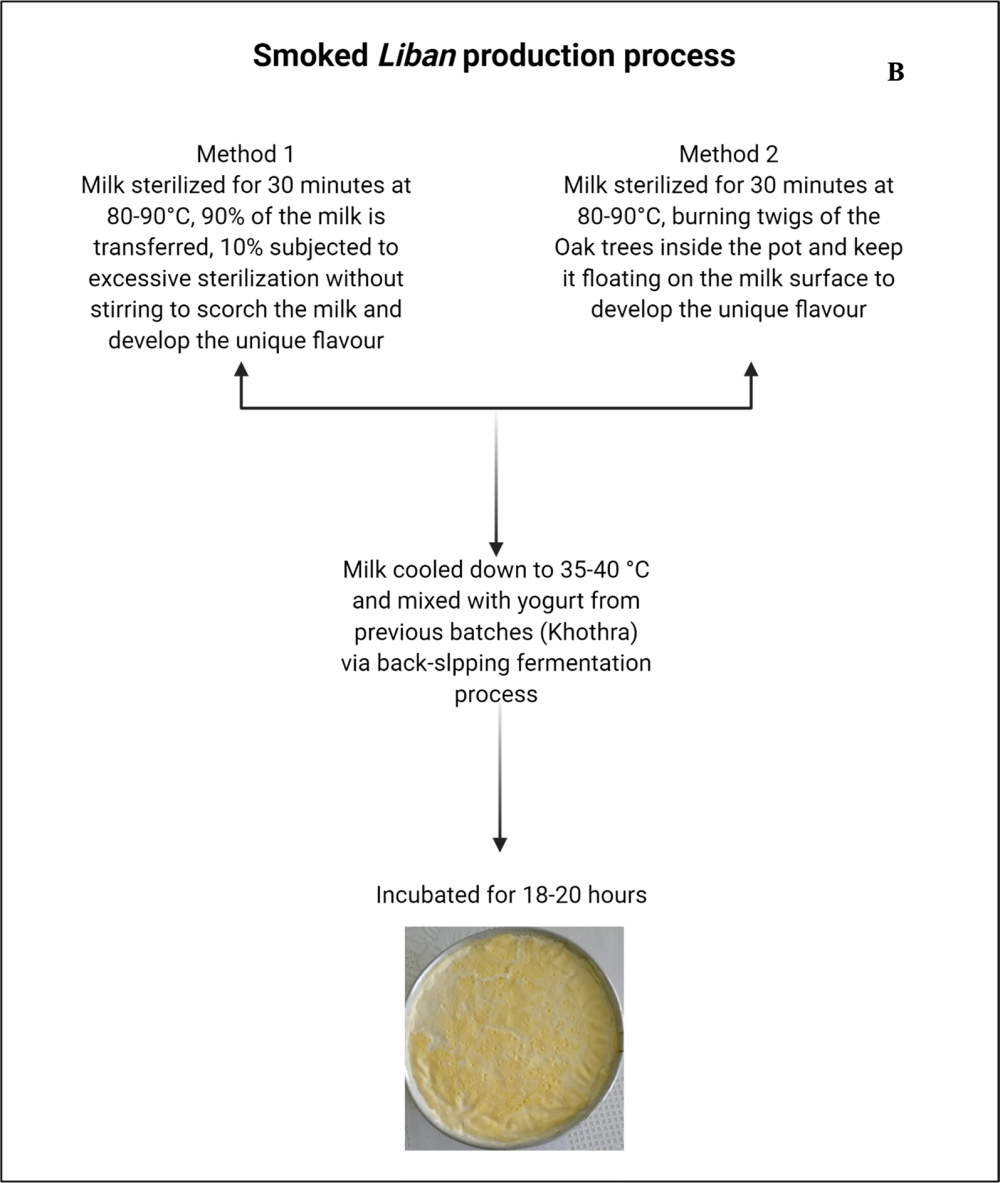

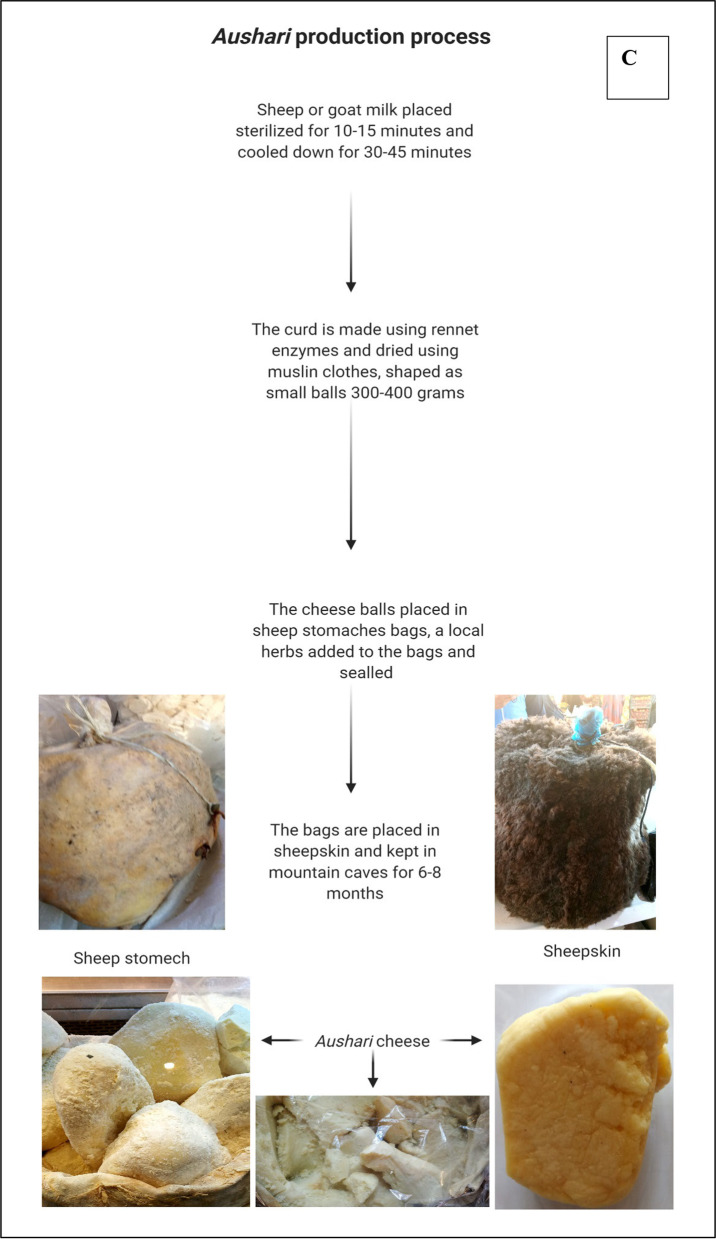

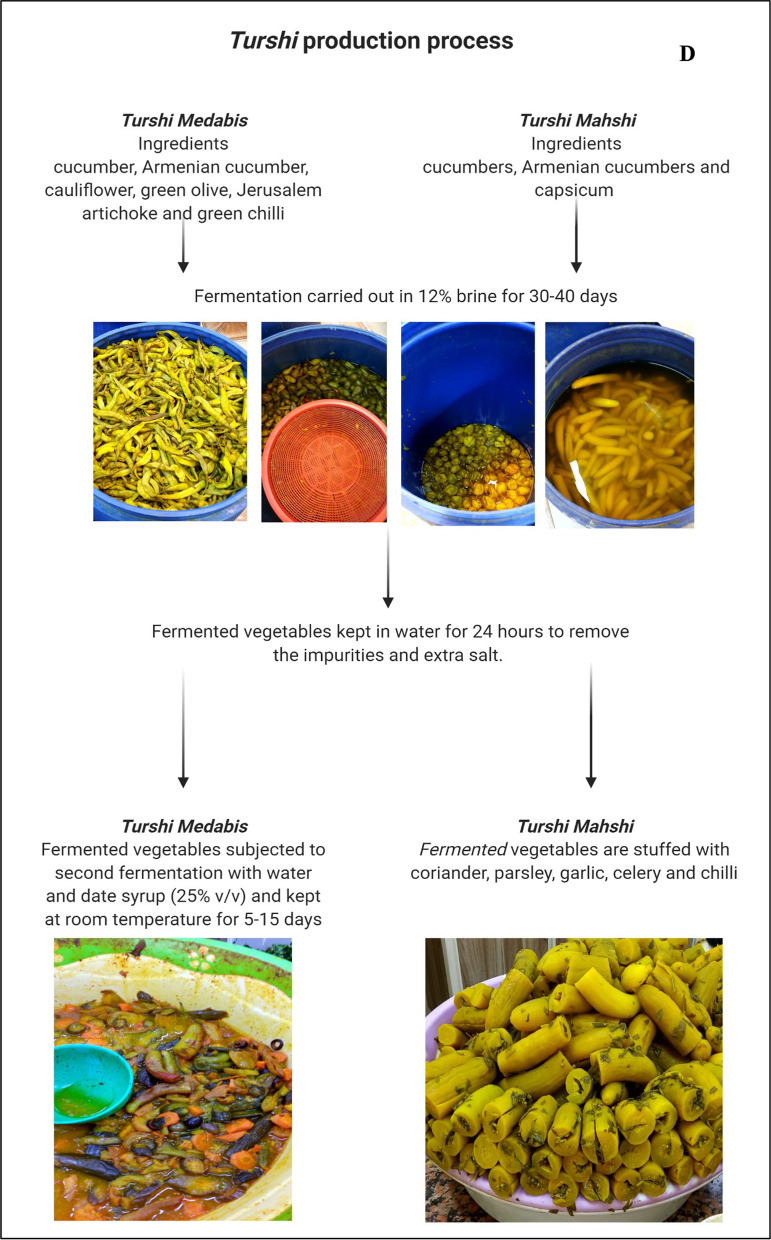

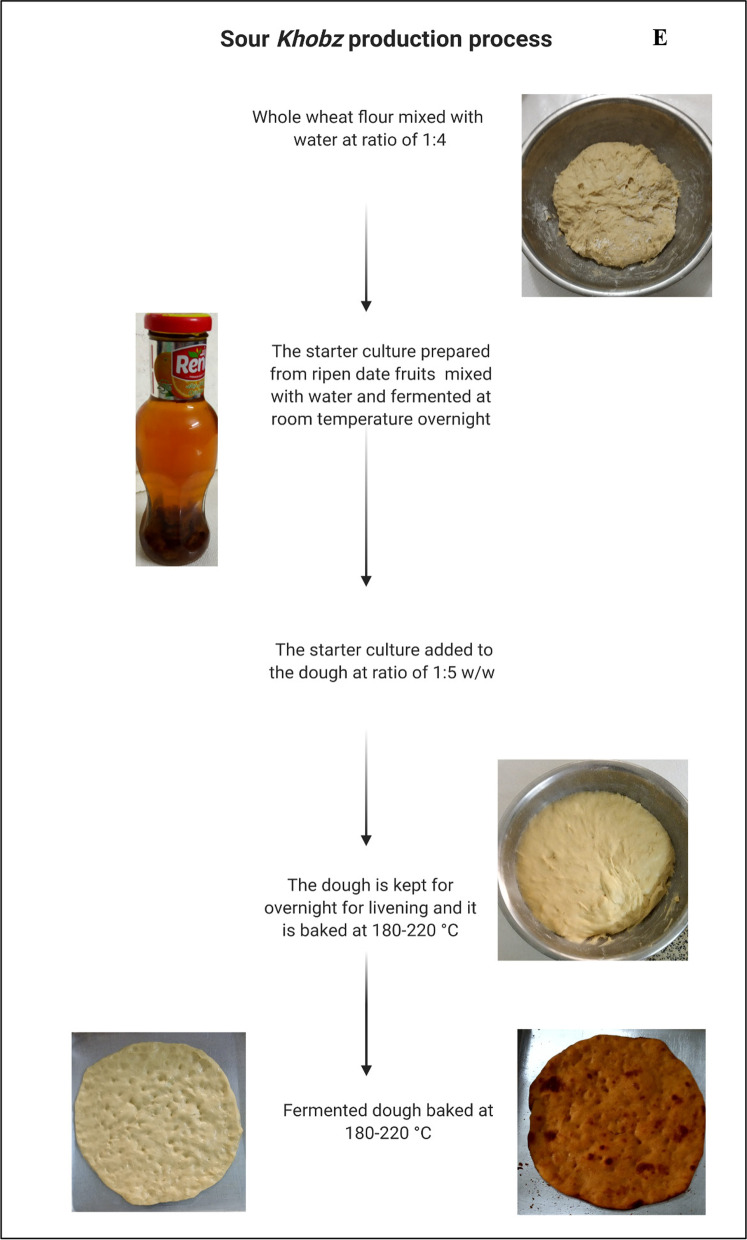
Production process for (**A**) *Basturma*, (**B**) smoked *Liban*, (**C**) *Aushari* cheese, (**D**) *Turshi*, (**E**) Sour *Khobz*

*Basturma* or *Bastirma* is traditional fermented meat prepared using minced lamb or minced beef that is mixed with several spices, salt, and garlic and stuffed in cow intestine [19]. The stuffed intestines are hanged on hooks and kept at room temperature for fermentation for 7–14 days. The traditional method for preparing *Basturma* is demonstrated in Fig. 2A. Minced beef or lamb should contain at least 10% fat as this significantly improves the aroma and enhances taste of the finished product. The finished product is safe to consume, and the quality remains stable for 7–10 days at 10–20 °C. After this period, it is recommended to freeze *Basturma* in order to maintain its quality and extend its shelf life.

*Basturma* is commonly consumed during the winter season after being fried in oil. It is consumed mainly during breakfast, and it is served with fried scrambled eggs. In recent years, the growing population of Iraq and high demand for *Basturma* has caused shortage of supplies for cow intestines used as casing. Synthetic casings are suggested as a safe alternative with added benefits of increased safety and reduced microbial contamination [20]. However, according to this study’s informants, consumers in Iraq refuse to use synthetic casing to produce traditional *Basturma*.

The challenge of preparing *Basturma* is safety as meat products are rich in nutrients which increases probability of contamination with *Salmonella* spp., *Staphylococcus aureus*, and *Escherichia coli* [21]. The microbial risk analysis for similar fermented food known as fermented sausage (salami) demonstrates low risk of contamination due to low water activity [22]. However, some studies have reported significantly higher risks of contamination of dry fermented meat products by several food-borne pathogens with the potential for outbreaks [23]. According to Ingham et al. [19], traditional *Basturma*, if prepared under strict hygiene control using meat from freshly slaughtered animals, offers non-significant risks of food-borne contamination and outbreaks. Therefore, the recommended production process for enhanced safety involves the implementation of strict hygienic conditions, utilization of fresh meat, and preservation of finished product by freezing.

### Smoked *Liban* (yogurt)

*Liban* (yogurt) is traditional fermented milk produced via the lacto-fermentation process using well defined starter cultures. The history of yogurt is not well known, but scholarly evidence indicates that yogurt has originated in the Mesopotamia at around 5000 BC. [24]. In several Middle Eastern countries, including Iraq, yogurt is known as the *Liban;* it is consumed with bread daily as a main dish for breakfast [25]. There are several varieties of *Liban* in Iraq that are traditionally prepared via back-slopping fermentation and made using different types of milks including cow’s, sheep’s, and goat’s milk. Traditional smoked *Liban* is unique for Iraq, and the most authentic product can be found in the north of Iraq, i.e. in the city of Erbil. Smoked *Liban* is well known among the Iraqi as the *Liban* Erbil due to its high quality and unique flavour.

Smoked *Liban* is prepared using sheep’s milk, i.e. milk of higher cost and quality compared to cow’s milk. According to this study’s informants, sheep’s milk is preferred by the locals due to its aroma. The *Liban* prepared from sheep’s milk has high physiochemical qualities and is rich in flavours because of high content of protein, solids, lipids, and polyunsaturated fatty acids [26]. Unique organoleptic properties of smoked *Liban* are due to a unique processing technique practiced by local producers (Fig. 2B).

There are two traditional methods to develop the unique flavour for smoked *Liban*. The first method is by sterilizing milk in a large copper pot; then, 90% of this milk is removed to smaller pots, and the other 10% is subjected to excessive sterilization without stirring. This is required to scorch milk and obtain a unique flavour. The second method to develop flavour is via burning twigs of oak trees inside the pot without touching milk. This sterilized milk is then cooled down to 35–40 °C and mixed with yogurt from previous batches of the best flavour known by local producers as the *Khothra*. *Khothra* is prepared from unsterilized fresh milk curd, and the curd is transferred (by the method of back-slopping) 3–4 times to develop the dominant starter cultures of preferred flavours. The technique was observed to improve quality and refine safety of dairy products by establishing the dominant starter cultures with antimicrobial and probiotic properties [12]. However, milk mixed with the yogurt from the previous batch is incubated for 18–20 h and sold fresh at local markets. The shelf life of the product is 7 days at room temperature, and 30 days at 4 °C. *Liban* is consumed by the local people in Iraq daily and mostly in the morning during breakfast.

### *Aushari* cheese

*Aushari* is traditional semi-hard cheese made of sheep’s and goat’s milk in the mountains of northern Iraq [27]. *Aushari* is mainly produced by the Kurdish tribes and can be found in the Kurdistan region, especially in Sulaymaniyah and Erbil. The villagers collect sheep’s or goat’s milk early in the morning, and milk is heated for 10–15 min and then cooled down for 30–45 min. This sterilized milk is used to prepare soft cheese using rennet enzymes from the fourth stomach of calves (abomasum). The curd is pressed using muslin clothes to remove the whey and reduce moisture content. The curd is shaped as cheese balls weighing 300–400 g each. The pressed soft cheese balls are placed in bags made of sheep stomach that have been pre-cleaned using water and salt. A traditional herb known locally as the *Jaje* belonging to the Thyme (*Thymus capitatus*) family is added to the bags to enhance flavour of *Aushari*. The bags are placed in sheepskin and transferred to the caves at the top of the mountains that have high humidity and low temperature. The cheese is matured for 6–8 months. During maturation, the cheese develops special aroma and taste. The ripening process is due to rennet enzymes, i.e. the enzymes from the sheep stomach, the enzymes from herbs, and natural microflora.

The cheese is commonly prepared in the summer and harvested from the caves during the winter. The ripened *Aushari* cheese has yellowish colour and a very strong, “sharp” aroma which is due to prolonged aging and added herbs (Fig. 2C). There are no accurate studies to profile the bioactive compounds and microbial load of *Aushari*. Generally, most aged cheeses are safe for consumption [28], but risk analysis of the *Aushari* cheese is yet to be performed. The cheese can be stored for 6 months at room temperature during the winter. The *Aushari* cheese is very popular with the Kurdish and less popular with other ethnic groups in Iraq.

### Turshi

*Turshi* is the most popular fermented food in Iraq. It is consumed daily and made of fermented mixed vegetables, such as cucumbers (*Cucumis melo* var. *flexuosus*), cauliflower (*Brassica oleracea* var. *botrytis*), cabbage (*Brassica oleracea* var. *oleracea*), green olive (*Olea europaea* L.), Jerusalem artichoke (*Helianthus tuberosus* L.), bell pepper (*Capsicum annuum*), and garlic (*Allium sativum*) [8]. There are two main preparation methods that can only be found in Iraq: the method from the city of Al-Najaf al-Ashraf which is known as the *Turshi Medabis* and the method known as the *Turshi Mahshi* from the historical city of Nineveh. *Turshi* is prepared via back-slopping fermentation using a portion of the previous batch that is added to the new batch. *Turshi Medabis* has a sweet–sour taste due to the mixture of flavouring ingredients including date syrup, apple cider, and tamarind.

A traditional recipe for preparing 10 kg of the finished product involves 3 kg cucumber, 2 kg cucumber, 2 kg cauliflower, 1 kg green olive, 1 kg Jerusalem artichoke, and 1 kg green chilli. Vegetables are washed and transferred to fermentation tanks to be covered with brine (12% salt). The first spontaneous fermentation is carried out for 30–40 days. The product is then kept in water for 24 h to remove impurities and extra salt. The fermented vegetables are subjected to second fermentation with water and date syrup (25% v/v) and kept at room temperature for 5–15 days.

*Turshi Mahshi* is prepared following the first fermentation process of *Turshi Medabis* using cucumbers and bell pepper only. In the second stage of fermentation, vegetables are stuffed with coriander, parsley, garlic, celery, and chilli. The stuffed vegetables are placed in a tank and fermented for 3–5 days with water containing 5% lemon juice and 10% date vinegar. The traditional Iraqi *Turshi* is served with a special condiment known as the *Amba* that is made by mixing pickled green mangoes, white vinegar, turmeric, fenugreek, and salt (Fig. 2D). *Amba* is, thus, a fermented condiment that is consumed with *Turshi* and as a sauce for sandwiches such as *Kebab* (minced beef grilled on charcoal) and *Falafal* (patties made of ground chickpeas).

*Turshi* is commonly consumed in Iraq as an appetizer during lunch and dinner, with consumption frequency increasing in the winter. Due to low acidity provided by lemon and vinegar, the product has long shelf life (10 days at room temperature). However, acidity can reduce quality and preferred texture of fermented vegetables. Therefore, *Turshi* is recommended to be consumed within 2–3 days. Burris et al. [29] report high risks of consuming cucumbers due to frequent outbreaks caused by *Salmonella enterica*. Cauliflower, cabbage, and bell pepper are also prone to outbreaks due to potential contamination with *Listeria* [30]. Recently, *Turshi* has been reported responsible for an outbreak of food-borne Botulism caused by *Clostridium botulinum* among the immigrants to the USA [31]. There are no studies determining safety of the traditional *Turshi* in Iraq. However, no outbreaks have been recorded due to consumption of *Turshi* for a long time. This can be a result of strict hygienic conditions maintained in local production facilities.

### Sour *Khobz*

Sour *Khobz* is traditional sour bread in Iraq made of whole wheat flour. The art of this bread’s production has been practiced for centuries by ancient civilizations in southern Iraq, such as the Babylon, Sumerian, Uruk, Lagash, and Ur. These ancient civilizations utilized date fruits as a starter culture for dough preparation because date fruits were believed to have exceptional health benefits [32]. Date fruits contributed to leavening the dough due to their significant microbial diversity including yeasts, acetic acid, and lactic acid bacteria [33]. Currently, this ancient art of bread preparation is rarely practiced nowadays. Lifestyle changes and the development of baking yeast have impacted on the production of the traditional sour *Khobz*. Importantly, bread made using date fruits or syrup can be found in several Arab countries including Bahrain where it is known as the *Khubez Tamer* [34]. Addition of date fruits to the dough has been reported as a novel method to extend shelf life and improve safety of sour bread [35].

The traditional production method is simple and begins by mixing whole wheat flour (1 kg) with 1 cup of water (250 mL) to prepare the dough. The starter culture is prepared by mixing ripen date fruits (10–15 pieces) with 250 ml water which is kept at room temperature overnight. The starter culture is added to the dough at the recommended ratio of 1:5 w/w to ensure good fermentation. The dough is kept overnight for leavening and baked in the oven at 180–220 °C to produce thick sour bread (Fig. 2E). This bread has long shelf life (6–8 days at room temperature) and improved quality with delayed staling. This bread is consumed in local communities of the Iraqi marshes (known as the Mesopotamian marshes) in southern Iraq.

## Traditional fermented beverages

The ancient civilizations of Iraq, especially the Sumerians, believed that fermented beverages made of barley, known as beer today, could bring joy and happiness [2]. Concurrently, these civilizations developed non-alcoholic fermented beverages made of raisins, milk, and herbs. Nowadays, these beverages can be found in different regions of Iraq. This section will describe these beverages alongside their preparation methods.

### Shanina (Shineena)

**Fig. 3:**
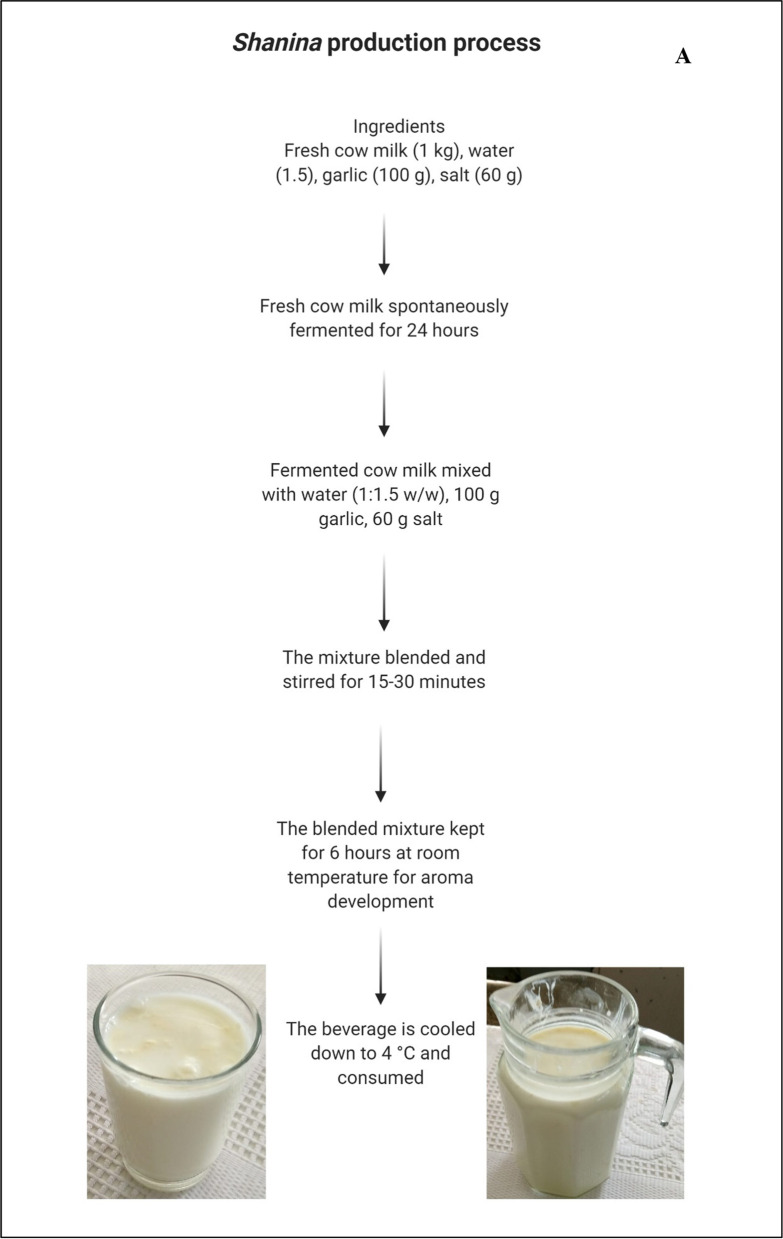

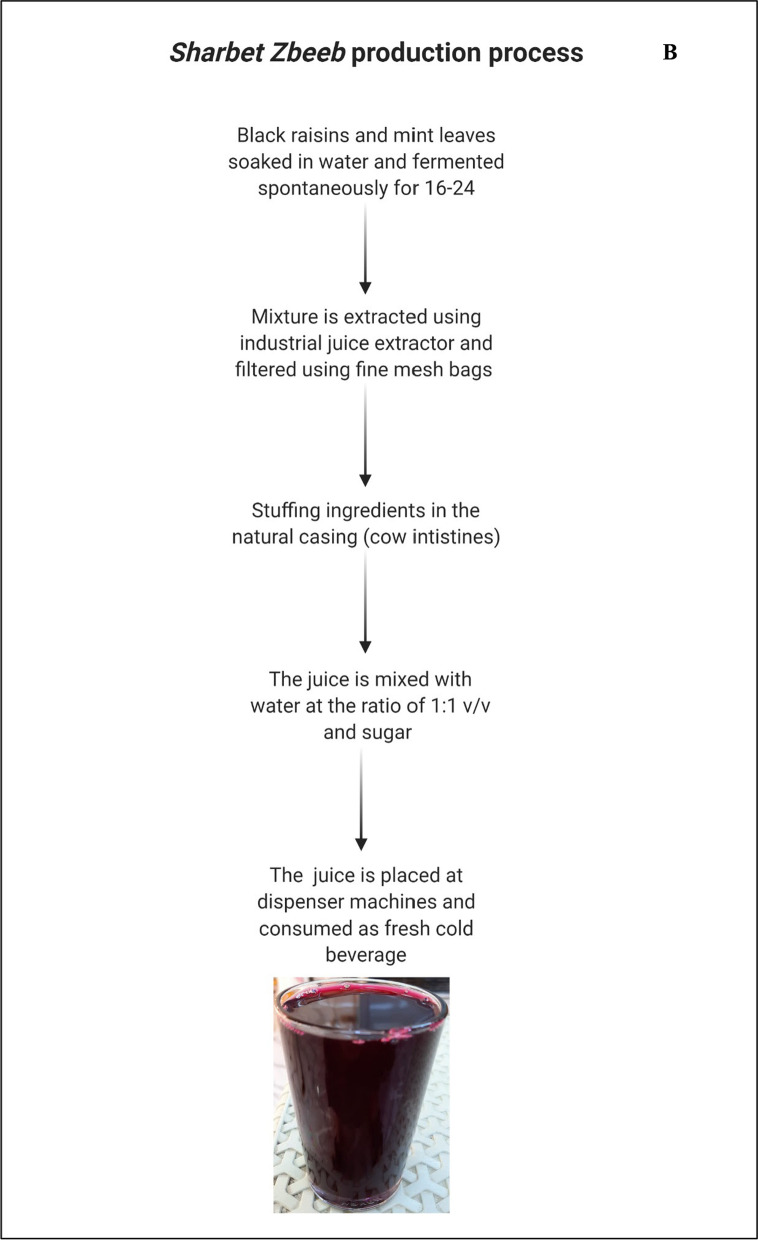

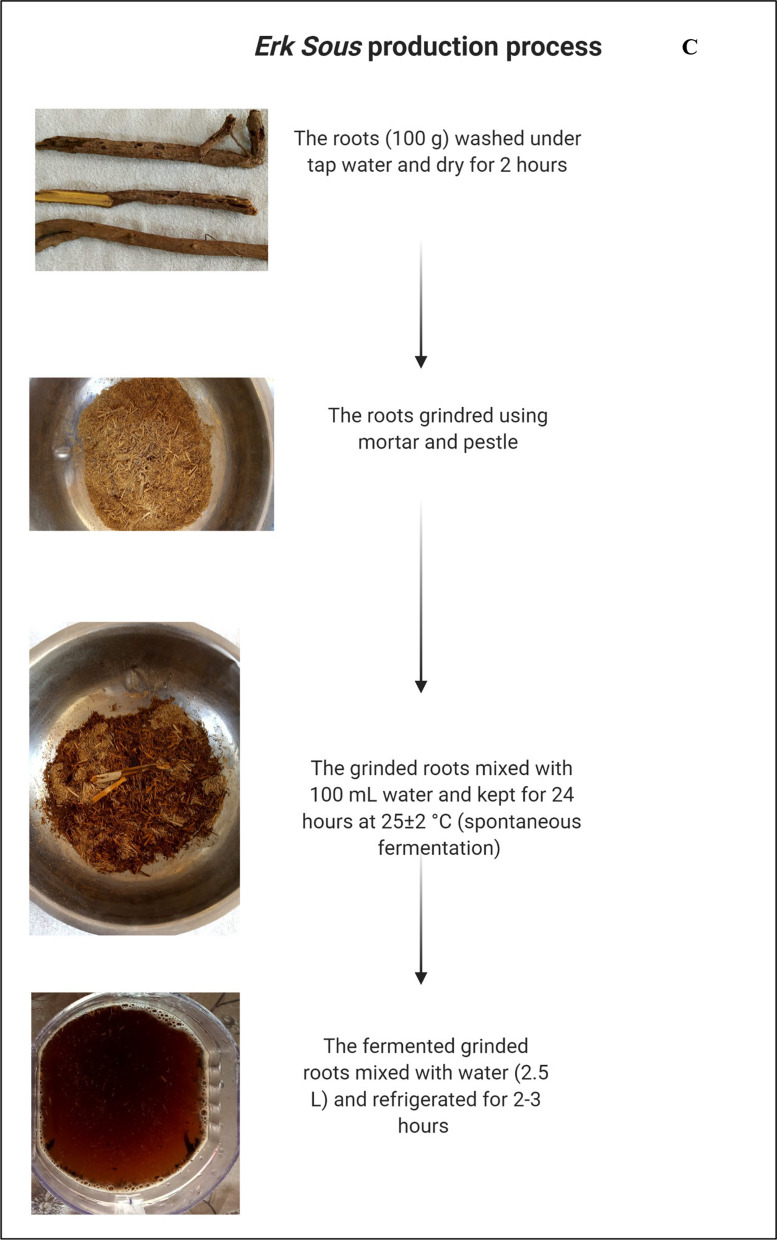
Production process for (**A**) *Shanina*, (**B**) *Sharbet Zbeeb*, (**C**) *Erk Sous*

*Shanina* is a fermented homemade yogurt beverage. This beverage has several versions in the Middle Eastern countries such as *Ayran* in Turkey [36] and *Doogh* in Iran [37]. These beverages are prepared by two methods: method one requires fermenting milk for a short time to form weak curd, while method two requires fermenting milk for a long time to prepare yogurt and then diluting it with water and adding salt. *Shanina* in Iraq is prepared via spontaneous fermentation of cow’s milk without thermal treatment for 24 h to prepare yogurt followed by mixing the yogurt with water at the ratio of 1:1 w/w with the addition of salt and garlic (Fig. 3A). The addition of garlic is unique to this product in Iraq as the other countries prepare it without garlic. Traditionally, the mixture is subjected to manual stirring for 10–15 min and the beverage is then kept for 6 h to develop aroma. The product is associated with several outbreaks in Iraq due to the absence of sterilization and/or heat treatment to inhibit growth of milk-associated pathogens. *Shanina* should be consumed within 24 h; otherwise, it will become very sour due to large microbial load. This increased sourness is due to lactic acid bacteria that produce lactic acid [38].

### Sharbet Zbeeb

*Sharbet Zbeeb* is fermented non-alcoholic juice that is consumed in Iraq as a refreshing beverage. The beverage is made of black raisins (*Vitis vinifera*) (1 kg) that are soaked in water (2 L) and kept being fermented spontaneously for 16–24 h with mint leaves (100 g). This sludge-resembling mixture is extracted using an industrial juice extractor and filtered using fine mesh bags. Filtered juice is then mixed with water at the ratio of 1:1 v/v and sugar (240 g). This fermented juice is placed in dispenser machines and subsequently consumed as a fresh cold beverage (Fig. 3B). Consumption of *Sharbet Zbeeb* dramatically increases during the fasting month of the Ramadan as this tradition is embedded in the Iraqis’ culture and has become part of their ritual.

*Sharbet Zbeeb* should be consumed within 48 h after fermentation due to increased sourness after prolonged storage. The beverage is stable towards microbial spoilage due to high phenolic compounds content found in raisins. Breksa III et al. [39] reported strong antioxidant activity of different raisins linked to high levels of quercetin, kaempferol, and rutin contents. Salih and Kadim [40] extracted a phenolic compound (Proanthocyanidin) from the traditional Iraqi *Sharbet Zbeeb* and demonstrated strong antimicrobial activity towards a broad range of food-borne pathogens. In addition, phenolic compounds content is significantly enhanced in *Sharbet Zbeeb* due to the presence of the mint leaves’ aqueous extract containing high amounts of phenolics [41].

The traditional method to prepare *Sharbet Zbeeb* is rudimentary and does not comply with international hygiene standards. This can endanger product safety due to high probability of contamination by several food-borne pathogens. Raisins have been reported to contain high levels of Ochratoxin A produced by several moulds [42]. Therefore, the implementation of the hazard analysis critical control point (HACCP) system is recommended to ensure safety of the produced beverage [43].

The traditional *Sharbet Zbeeb* is considered a “ready to drink” beverage that is not subjected to treatment after fermentation and contains various live cultures. Therefore, the beverage develops high acidity within 24–48 h of storage at chilling temperature resulting in low quality with potential safety issues. Thermal treatment and sterilized bottle filling can improve quality and extend shelf life of the beverage. When produced in compliance with international hygiene standards, this traditional beverage can be exported to neighbour countries, including Jordan, Turkey, Iran, Kuwait, Saudi Arabia, and Syria.

*Sharbet Zbeeb* is considered a natural fruit juice similar to blackcurrant, blackberry, raspberry, strawberry, blueberry, and cranberry beverages. These are globally commercialized as natural fruit juices with high antioxidant activity and health benefits due to their outstanding phenolic compound content [44]. *Sharbet Zbeeb* is produced via fermentation which can enhance its health benefits due to more active release of phenolics from fruits via enzymatic activity [45]. Therefore, *Sharbet Zbeeb* may have significant potential to penetrate the global market of beverages especially in light of growing consumer demand for healthy, unprocessed foods and beverages.

### Erk Sous

*Erk Sous* is a traditional Iraqi beverage prepared using the roots of liquorice (*Glycyrrhiza glabra*) via spontaneous fermentation. The beverage is consumed in the summer as a refreshment drink and in the winter as an energy drink. Popularity of *Erk Sous* in the Iraqis’ culture is due to the abundance of liquorice plant at the water rims of the Tigris and Euphrates. Liquorice roots were found on clay tablets from the Babylon civilization and in the Assyrian Empire [46]. Liquorice roots have several food applications such as in bakery, dairy, meat products, but also as sauces, candies and non-alcoholic beverages [47].

To produce *Erk Sous,* liquorice roots are mixed with water at the ratio of 1:1 and subjected to milling to form a paste using mortar and pestle. The paste is then kept in a pottery jar in darkness for 2 days. The paste is soaked in water overnight, and the resultant juice is filtered using muslin cloth (Fig. 3C). The beverage is very stable towards spoilage and physiochemical changes for 4–8 days without any treatment. Liquorice extract has been utilized as an ingredient for preparing a fermented sugared tea beverage (known as Kombucha) in combination with black and green tea [48]. Liquorice aqueous extract contains high amounts of different carbohydrates that may improve organoleptic properties of Kombucha [49].

## Commercial potential and limitations

Fermented foods and beverages are well known for their health benefits due to high content of bioavailable nutrients and probiotics [50]. Fermented foods play an important socio-economic role in developing countries by making a major contribution to public nutrition. The fermentation process can enhance safety of foods and beverages by degrading any mycotoxins from fungi that can be found in raw material [51]. Fermentation can preserve and extend shelf life of food products that are only available during specific seasons. Fermentation can thus better valorize fresh produce and reduce food loss and waste during harvesting [52]. Further advancements in fermentation technology will strengthen local and regional food security and increase employment opportunities at the stages of growing food (agriculture) and making food (food manufacturing).

Traditional fermented foods and beverages of Iraq are derived from various ancient civilizations and produced via well-established fermentation techniques. This showcases safety of traditional foods and beverages, thus signalling the potential for large-scale commercialization. Local food manufacturers in Iraq have been producing traditional fermented foods and beverages for centuries and developed reputation and even brands that are well known locally. This has become possible in part due to low operational costs. For example, the workforce is available in Iraq at a monthly average salary of 400 USD. Raw material to produce traditional foods and beverages is abundant and available at a low cost throughout the year.

The limitations of locally produced fermented foods and beverages in Iraq include inadequate knowledge and implementation of food hygiene, absence of modern production and storage technologies, lack of advanced packaging techniques, and limited certification agencies to meet international food quality standards. Local producers of traditional foods and beverages in Iraq do not possess marketing knowledge which is detrimental to commercialization. The power of multinational corporations (MNCs) reduces the potential of traditional foods and beverages to spread outside Iraq. For example, manufacturers of soft drinks, such as Coca-Cola and PepsiCo, have established their presence across the world and their products replace traditional beverages due to aggressive marketing. However, growing consumer demand for healthy foods and beverages can drive commercialization of traditional fermented foods and beverages which are healthier than many globally known brands [53]. This demand has grown due to COVID-19, and many studies have demonstrated the role of traditional fermented foods and beverages in enhancing immunity [54]. The consumption shift towards healthy lifestyles can accelerate commercialization of traditional fermented foods and beverages of Iraq, especially in countries that share common eating/cultural habits, such as those located in the Middle East, but also in Eastern Europe, North Africa, and Southeast Asia.

However, in developed countries, fermented foods and beverages are not commonly consumed and/or commercialized at an industrial scale [55]. Despite their health benefits, fermented foods and beverages in developed countries are replaced with modern processed foods and/or foods and beverages manufactured by MNCs. Although processed foods are convenient in preparation, tasteful due to additives, and have extended shelf life of 24–36 months [56], traditional foods and beverages can appeal to the modern consumer due to their healthiness. If effective marketing is applied, traditional foods and beverages can provide an investment opportunity even in the well-established food consumption markets of Western Europe and North America. Growing popularity of quinoa, the traditional food of the Andes, especially among the younger consumer segments [57], provides an encouraging example for traditional foods and beverages of Iraq.

The restrictions to import fermented foods by the Food and Drug Administration of the USA and the European Food Safety Authority of the European Union have limited the spread of fermented foods and beverages in developed countries. Further, most fermented foods and beverages produced in developing countries suffer from ineffective packaging with resultant short shelf life. This complicates the logistics of fermented foods and beverages from production to consumption. However, several fermented foods and beverages have been exported from developing countries to developed countries and become highly accepted and consumed such as Kimchi [58] and Tempeh [59]. The COVID-19 pandemic has significantly influenced consumer perception towards the health benefits of fermented foods and beverages. This shift presents an opportunity for small- and medium-scale producers in Iraq and other developing countries to expand their markets. The health benefits of fermented foods and beverages can prompt consumers to improve their well-being in the face of the recent and future pandemics.

## Conclusion

Traditional fermented foods and beverages are derived from various ancient civilizations, and their preparation techniques have been inherited over centuries from one generation to another. Although traditional fermented foods and beverages of Iraq originate from ancient diets, they remain popular nowadays among the local people. However, no research has attempted to document these traditional fermented foods and beverages and explore their production techniques. No study has been undertaken to assess the risk of traditional fermented foods and beverages in Iraq to public health and their association with outbreaks. This is a major shortcoming as traditional fermented foods and beverages of Iraq have significant commercialization potential commercialization given their health benefits and pleasant taste.

This current study has reviewed traditional fermented foods and beverages of Iraq. By employing the method of ethnographic research, the study has documented the main production techniques. By supplementing findings with the literature review, the study has also shed light on safety of traditional foods and beverages in Iraq. Lastly, the study has briefly discussed the potential for traditional Iraqi foods and beverages to upscale their production.

In terms of future research outlook, studies should aim at identifying the acceptable levels of food safety and hygiene required to prepare traditional foods and beverages and establish the data on their microbiology loads. Moreover, the implementation of the HACCP system will significantly improve safety of fermented foods and beverages of Iraq. Research should also be carried out to profile bioactive compounds and evaluate health benefits of traditional fermented foods and beverages. Studies are also necessitated to determine how traditional fermented foods and beverages of Iraq can effectively compete with modern foods and beverages, especially from the perspective of their perceived healthiness and marketing and particularly in light of COVID-19. Consumption habits of different generations, particularly Generation Y and Z, should be investigated given that these consumer groups are characterized by higher health concerns. A cost benefit analysis of producing traditional foods and beverages in Iraq should also be conducted to calculate the expense of their prospective commercialization.

## Data Availability

Not applicable.
